# A Simplified Model of Choice Behavior under Uncertainty

**DOI:** 10.3389/fpsyg.2016.01201

**Published:** 2016-08-17

**Authors:** Ching-Hung Lin, Yu-Kai Lin, Tzu-Jiun Song, Jong-Tsun Huang, Yao-Chu Chiu

**Affiliations:** ^1^Department of Psychology, Soochow UniversityTaipei, Taiwan; ^2^Department of Psychology, Kaohsiung Medical UniversityKaohsiung, Taiwan; ^3^Research Center for Nonlinear Analysis and Optimization, Kaohsiung Medical UniversityKaohsiung, Taiwan; ^4^Biomedical Engineering Research and Development Center, China Medical University HospitalTaichung, Taiwan; ^5^Graduate Institute of Neural and Cognitive Sciences, China Medical UniversityTaichung, Taiwan

**Keywords:** Iowa Gambling Task, expected utility model, prospect utility model, dynamic-uncertainty situations, gain-loss frequency, loss aversion, delta learning rule, prominent deck B phenomenon

## Abstract

The Iowa Gambling Task (IGT) has been standardized as a clinical assessment tool (Bechara, 2007). Nonetheless, numerous research groups have attempted to modify IGT models to optimize parameters for predicting the choice behavior of normal controls and patients. A decade ago, most researchers considered the expected utility (EU) model (Busemeyer and Stout, 2002) to be the optimal model for predicting choice behavior under uncertainty. However, in recent years, studies have demonstrated that models with the prospect utility (PU) function are more effective than the EU models in the IGT (Ahn et al., 2008). Nevertheless, after some preliminary tests based on our behavioral dataset and modeling, it was determined that the Ahn et al. (2008) PU model is not optimal due to some incompatible results. This study aims to modify the Ahn et al. (2008) PU model to a simplified model and used the IGT performance of 145 subjects as the benchmark data for comparison. In our simplified PU model, the best goodness-of-fit was found mostly as the value of α approached zero. More specifically, we retested the key parameters α, λ, and A in the PU model. Notably, the influence of the parameters α, λ, and A has a hierarchical power structure in terms of manipulating the goodness-of-fit in the PU model. Additionally, we found that the parameters λ and A may be ineffective when the parameter α is close to zero in the PU model. The present simplified model demonstrated that decision makers mostly adopted the strategy of gain-stay loss-shift rather than foreseeing the long-term outcome. However, there are other behavioral variables that are not well revealed under these dynamic-uncertainty situations. Therefore, the optimal behavioral models may not have been found yet. In short, the best model for predicting choice behavior under dynamic-uncertainty situations should be further evaluated.

## Introduction

The Iowa Gambling Task (IGT) is an experience-based decision-making task used extensively as a diagnostic tool for neuropsychiatric disorders (Bechara et al., 1994, 1997). It can identify various psychological disorders, such as schizophrenia and substance addiction (Bechara, 2007). Following the logic of IGT development, researchers have attempted to discover the predictors and explored the mechanisms of emotional systems and decision-making functioning under situations of uncertainty. In the IGT, decks A and B have a negative final outcome (that is, a long-term outcome of -$250) over an average of 10 trials. Conversely, decks C and D have a positive final outcome (+$250) over an average of 10 trials. According to these standard final outcomes, decks A and B are termed “bad decks” and decks C and D are termed “good decks.” At the same time, decks B and D contain infrequent losses (10 gains and 1 loss over 10 trials) while decks A and C contain relatively frequent losses (10 gains and 5 losses over 10 trials). IGT-related neuropsychological studies have mostly demonstrated that patients (e.g., individuals with ventromedial prefrontal lesions) prefer to choose the bad decks to a greater degree than do healthy controls. However, in recent years, some IGT studies have demonstrated that healthy controls also prefer to choose the bad deck B due to its frequent gains (Wilder et al., 1998; Steingroever et al., 2013), a finding which has been called the “prominent deck B phenomenon” (Lin et al., 2007; Chiu et al., 2012).

The expected utility (EU) theory (von Neumann and Morgenstern, 1947) has been used most frequently over the years to predict choice behavior. The original assumption and design of the IGT was based mainly on extensions of the EU theory (Bechara et al., 1994, 1997; Bechara and Damasio, 2005). However, studies on behavioral decision-making over the past five decades (Edwards, 1954; Tversky and Kahneman, 1986; Kahneman, 2003) have indicated that a decision maker's choice is not guided by the EU, but mainly by information regarding gain and loss under risk, as suggested by Prospect Theory (PT) and the Framing Effect. Prospect Theory demonstrated that most decision makers prefer to take risks in situations with negative descriptions (e.g., loss and death) and become risk-averse in situations with positive descriptions (e.g., gain and life) (Kahneman and Tversky, 1979; Tversky and Kahneman, 1981). Experiments in PT showed that normal decision makers ignored the EU and that their attitudes toward risks varied according to the depiction of the situation. This finding goes completely against the traditional viewpoints of economics and rationales in terms of invariant axioms (Kahneman and Tversky, 1979; Tversky and Kahneman, 1981, 1986). However, these studies were based mostly on description-based rather than dynamic-consecutive (experience-based) games such as the IGT (Barron and Erev, 2003; Hertwig et al., 2004; Hau et al., 2008; Fantino and Navarro, 2012).

Behavioral modeling is efficient for interpreting behavioral results and predicting differences in choice patterns between normal decision makers and neuropsychiatric patients. Many IGT modeling studies have indicated that modeling based on the EU theory is sufficient for distinguishing between neuropsychiatric patients (or criminals) and healthy subjects (Busemeyer and Stout, 2002; Garavan and Stout, 2005; Stout et al., 2005; Yechiam et al., 2005; Luo et al., 2011). However, some alternative theories, such as the viewpoint based on gain-loss frequency, have also been used to interpret the choice behavior in such dynamic-uncertain games (Wilder et al., 1998; Lin et al., 2007; Chiu et al., 2008; Upton et al., 2012). Furthermore, based on the profound finding of Ahn et al. (2008), more and more behavioral-modeling studies have demonstrated that the PT-related models (which consider the effects of both gains and losses, or PU function, in their modeling) are more predictive than EU models (Ahn et al., 2008; Fridberg et al., 2010; Horstmann et al., 2012; Worthy et al., 2013a,b; Worthy and Maddox, 2014; Dai et al., 2015). In short, these modeling studies have consistently indicated that the prospect of an immediate gain-loss is an important guiding factor in the choice of behavior in the IGT (Lin et al., 2004, 2007; Chiu et al., 2005, 2008):

“*Subjects may apply an implicit strategy to cope with the uncertain game, therefore they favored high-frequency gains over high-frequency losses in the experiment. This “gain-stay, lose-randomize” strategy (**Figure 3**) [42] has been observed in human and animal appetitive and avoidance experiments in which human or animal encounter reward or punishment [42–48].” (Chiu et al., 2008, p. 5)*.

To explain their results, most of these IGT-PU models (e.g., Yechiam and Busemeyer, 2005, 2008; Ahn et al., 2008; Fridberg et al., 2010; Horstmann et al., 2012; Steingroever et al., 2014; Worthy and Maddox, 2014; Dai et al., 2015) were modified from the EU models or were hybrid models combining the PU function with general behavioral learning models such as the Prospect Valence Learning (PVL) model (Ahn et al., 2014), and learning rules such as the delta learning rule, DEL (see Ahn et al., 2008, p. 1384, Equation 3; Rescorla and Wagner, 1972) and the decay reinforcement learning rule, DRI (see Ahn et al., 2008, p. 1385, Equation 4; Erev and Roth, 1998).

For instance, to determine an optimized decision model under dynamic-uncertainty, Ahn et al. (2008) compared eight decision-learning models with regard to their generalizability. Each decision maker took part in two dynamic-uncertain games, namely the IGT and the Soochow Gambling Task (SGT) (Chiu et al., 2008). Data from the first game was used to estimate the parameters of each model and to make a prediction for the second game. Furthermore, Ahn et al. (2008) adopted three methods to evaluate the goodness-of-fit of each model for each participant: a *post-hoc* fit criterion, a generalization criterion for short-term estimations, and a generalization criterion for long-term estimations. Consequently, they suggested that the PU function provides the optimized predictions for new conditions, but different learning models are needed to make short- vs. long-term learning predictions.

However, these refined PU models were relatively complex, compared to the original PU function (Kahneman and Tversky, 1979; Tversky and Kahneman, 1992). Recent studies in behavioral modeling have suggested that a simplified model based on the gain-stay loss-shift (or win-stay lose-shift) principle may provide a sufficient explanation of choice behavior under uncertainty (Lin et al., 2007; Chiu et al., 2008, 2012; Worthy et al., 2013a,b). Moreover, some research has suggested that these simplified models could be consistent with a large number of behavioral studies:

“*These pioneer behavior studies with the concurrent schedules of reinforcement have displayed the frequency effect for choice pattern [45–49]. Additionally, these concepts have also been applied to examine the behavioral model of neuropsychological deficit [50,51].” (Chiu et al., 2008, p. 5)*.

The purpose of this study is to simplify the structure of the PU model and to provide the behavioral modeling to test the effects of some parameters. Additionally, this study identifies which type of parameter modulation has an optimal goodness-of-fit for the behavioral data. Based on the original assumption of the PU function and the findings of recent gain-loss frequency studies, we hypothesized that if the choice behavior of normal decision makers is based mostly on the gain-stay loss-shift (win-stay lose-switch) strategy, the optimized behavioral model should be relatively simpler than that which Ahn et al. (2008) had proposed. Namely, the weighting power of immediate gain-loss should be larger than the learning effect of gaining long-term outcome. (Note: For the original form of the equations and the method of simulation adopted in the present study, please refer to Ahn et al. (2008).)

## Materials and methods

### Participants

We recruited 145 participants who were all college students (102 males and 43 females, mean age: 18.6, SD: 0.97). Most of the subjects were first-year students. All statistical data was analyzed at a group level and presented anonymously. In this study, the participants were welcome and totally free to participate in the psychological experiment in the university, and the procedure was consistent with publicly available literature. After completing the whole game, the authors provided a 2-h lecture on human decision-making behaviors that also explained the testing purpose and the psychological mechanism of the IGT for all the subjects. The behavioral data was collected in October 2010, at which time the Institutional Review Board approval system was still in the process of being implemented at our university. The study was conducted in accordance with the unwritten rules of the Taiwan Psychological Association. Further, the IGT was conducted to simulate real-life decisions, so it looked like a common computerized card game that someone might play on the Internet. Many research websites provide online versions of the IGT to recruit general participants via the Internet (such as http://www.millisecond.com/download/samples/v3/IowaGamblingTask/IowaGamblingTask.web and http://pebl.sourceforge.net/battery.html). Simply put, anyone can play the online version of IGT totally free.

### Materials

**Figure 1:**
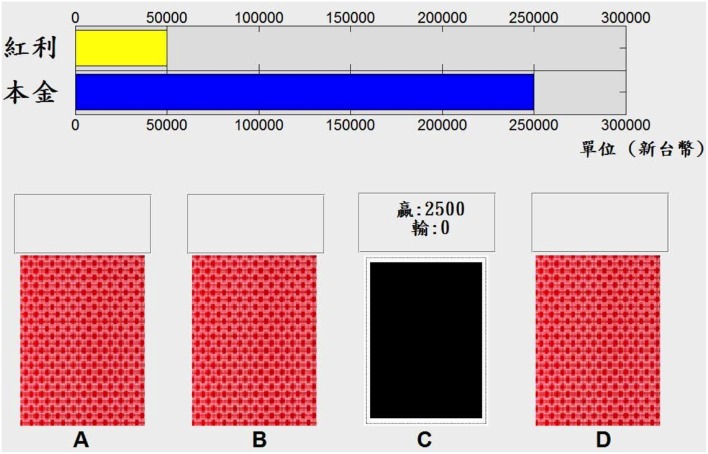
**Appearance of the computerized version of the Iowa Gambling Task (IGT) used in this study**. The computerized version of the IGT used in this study had most of the characteristics of the original IGT design. However, it used the Chinese language for participants in Taiwan increases the monetary value to fit the New Taiwan Dollar currency. The blue bar represents money borrowed from the bank and the yellow bar represents extra money (bonus) in this task.

The gain-loss structure of the IGT in this study followed the original table outlined by Bechara et al. (1994), and the computerized version of the IGT was programmed with Matlab 2010a (MathWorks, Natick, MA, USA). Figure 1 shows the appearance of this computerized version.

### Procedures for collecting behavioral data

The original instruction of the IGT was adopted in this study. At the beginning of the game, the instructor provided instructions to ensure that the participants knew how to play this game. Each participant had a 200-trial selection. Participants used a mouse to pick a desired deck and the screen displays “win money” or “lose money” immediately, and the outcomes are summarized in the top bars (Figure 1). The participants did not know when the game would terminate. They were asked to do their best to earn money or avoid losing money in the IGT, but the instructor provided no hints for success in this task. Each participant in the present study played a game consisting of 200 trials, but only the dataset for the first 100 trials was used as the comparison data. This use of the data from the first 100 trials is comparable to the standard approach used for administering the IGT in past IGT-related studies. Meanwhile, the dataset for the last 100 trials for each participant was not analyzed in this study. The dataset for the last 100 trials could be valuable, however, in future studies aimed at exploring the extended learning effect.

### Procedures for producing simulation data

A simulation method (see Ahn et al., 2008, p. 1401, Appendix B) was used to estimate the parameters (Yechiam and Busemeyer, 2005; Ahn et al., 2008), with a few initial steps modified. First, the behavioral datasets for all participants were averaged and inserted into the model. Here we averaged the behavioral data across subjects to reduce the variance in the individual results; specifically, we used the mean probability of each deck chosen as the initial index during simulation. Second, according to the results of the eight models in Ahn et al. (2008), the models with the PU function (see Ahn et al., 2008, p. 1384, Equation 2) were proven to be better than those with the EU model. In summary, Ahn et al. (2008) applied the PU function to decision-learning models (DEL and DRI) and showed that the PU models are more powerful than EU models for achieving optimized simulation results. They also showed that the mean square deviation (MSD) of the DEL model was relatively small, in comparison with the DRI model (see Ahn et al., 2008, p. 1392, Table 6). The original formula suggested by Ahn et al. (2008) is as follows [see p. 1384, Equations (2) and (3)].

PU model (PU-DEL learning model):

$$\begin{array}{l} {\text{E}}_{\text{j}}(\text{t})\;={\text{E}}_{\text{j}}(\text{t}-1)+\text{A}\cdot \delta_{\text{j}}(\text{t})\cdot [|\text{x}(\text{t}){|}^{\alpha }-{\text{E}}_{\text{j}}(\text{t}-1)],\text{ if x}(\text{t})\geq 0;\\ {\text{E}}_{\text{j}}(\text{t})=\text{​ }{\text{E}}_{\text{j}}(\text{t}-1)+\text{A}\cdot \delta_{\text{j}}(\text{t})\cdot [-\lambda |\text{x}(\text{t}){|}^{\alpha }\text{​}-{\text{E}}_{\text{j}}(\text{t}-\text{​ }1)],\text{if x}(\text{t})\text{ ​}<\text{ ​}0. \end{array}$$

In the above formula, E_j_(t) refers to the expectancy for deck j on trial t, A is the updating parameter, and δ_j_(t) a dummy variable which is 1 if deck j is chosen and 0 otherwise. In addition, x(t) symbolizes the net gain on trial t, λ represents a loss-aversion parameter, and α is defined as a shape parameter of the utility function (Ahn et al., 2008).

In this study therefore, we used only the best learning models in Ahn et al. (2008). In the first step of the preliminary test, we used two general approaches: the general simulation method and the one-step-ahead method (see Ahn et al., 2008, p. 1400, Appendix A). Applying the general simulation method, the chance level probability of each deck being chosen in the first trial is used (25% in the case of four decks). Therefore, the first trial is randomly produced. Given the result of the first trial, the selection probability of the following trials can be determined using the default initial values (see Ahn et al., 2008, p. 1385, Equation 5). For instance, if j represents one of the four decks, and j = 1 corresponds to deck A, then Pr_1_(2) marks the probability of deck A in the second trial, whilst E_1_(1) marks the expectancy of deck A in the first trial. Hence, the probability of each deck can be determined. Conversely, the one-step-ahead approach was totally dependent on the empirical data. Specifically, feeding real data from each trial into the model generated the probability of each following trial. These two approaches integrated the DEL model (Rescorla and Wagner, 1972; Yechiam and Busemeyer, 2005, 2008; Ahn et al., 2008) and the DRI model (Erev and Roth, 1998; Yechiam and Busemeyer, 2005, 2008; Ahn et al., 2008, 2014; Luo et al., 2011) for the final simulation, and the optimal parameters (α, λ, and A) were evaluated by MSD (see Ahn et al., 2008, p. 1391, Equation 11). Consequently, we found that the best result was obtained by using the model of general simulation combined with the DEL model. This result is mostly consistent with the observation by Ahn et al. (2008, see p. 1392, Table 6). The result of the preliminary test is listed in Table 1.

**Table 1:** **Comparison of the optimized parameter values of DEL and DRI models**.

**Evaluation method**	**Learning model**	**MSD**	**A**	**λ**	**α**
General simulation method	DEL	0.015131	0.1	1.3	0
General simulation method	DRI	0.017857	1	1.2	0.9

### Why is parameter C removed first?

**Figure 2:**
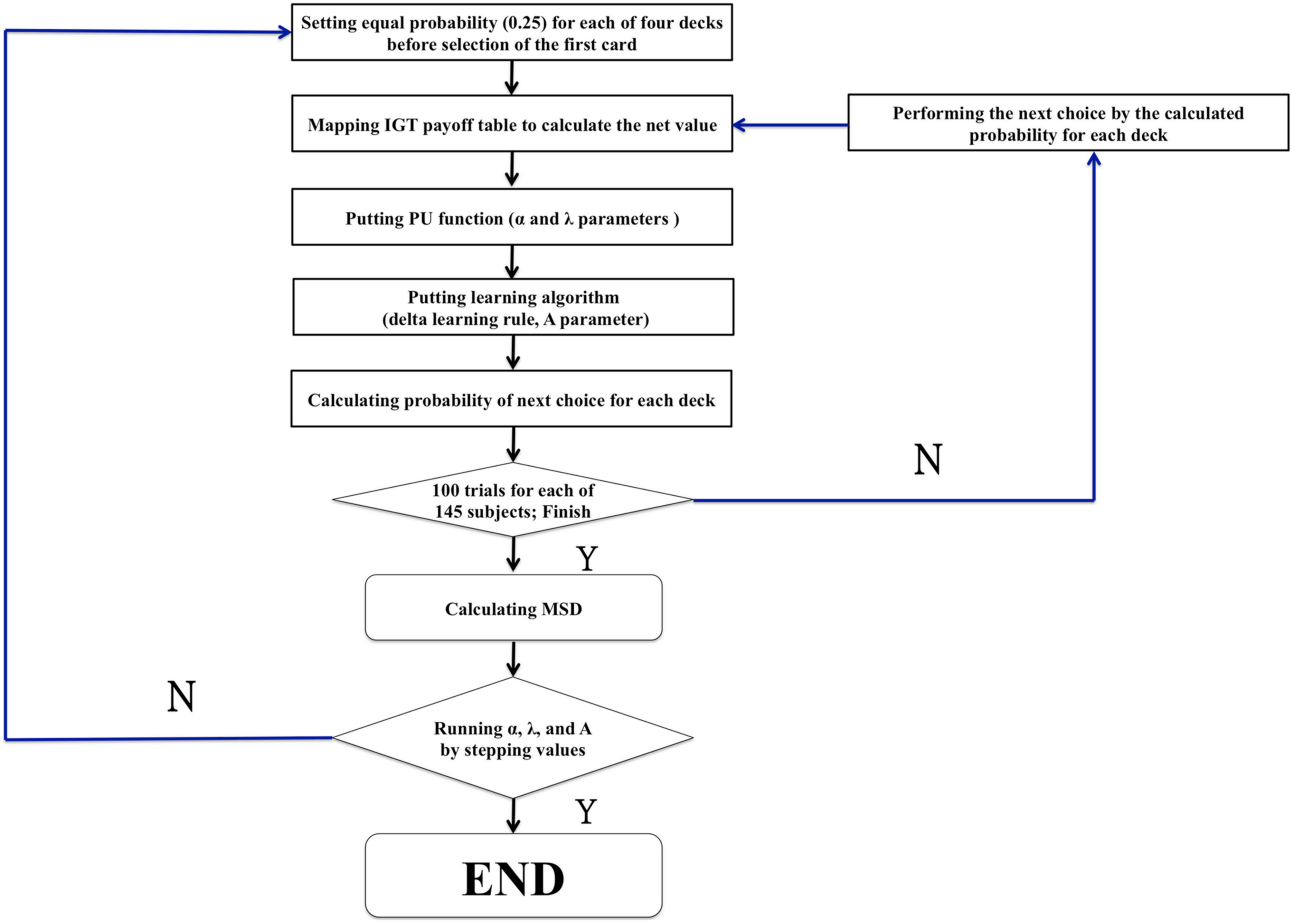
**The modeling procedure of the present study**. The flowchart shows the common procedure for performing the modeling study. The general game rule with random choice was first launched to simulate the initial stage when performing the IGT. However, the present study reexamines the power of influence of some critical parameters (α, λ, and A) in the PU model. There were 1936 rounds (11 × 16 × 11 = 1936) (α: 11 values [0, 0.1, 0.2, 0.3, 0.4, 0.5, 0.6, 0.7, 0.8, 0.9, 1.0]) (λ: 16 values [1.0, 1.1, 1.2, 1.3, 1.4, 1.5, 1.6, 1.7, 1.8, 1.9, 2.0, 2.1, 2.2, 2.3, 2.4, 2.5]) (A: 11 values [0, 0.1, 0.2, 0.3, 0.4, 0.5, 0.6, 0.7, 0.8, 0.9, 1.0]).

The c parameter (see Ahn et al., 2008, p. 1386, Equation 6) is defined as the consistency between choices and expectancies and is known as the response-sensitivity parameter (Yechiam et al., 2005). However, this parameter c was designed for EU-based models and to modulate the differences between the behavioral data and EU model predictions. Therefore, we consider it can be ruled out in our model because the present study was mostly based on the PU models of Ahn et al. (2008) in which the response-sensitivity parameter is removed, whilst virtual decision-making responds directly to parameters α, λ, and A. Therefore, the parameters were defined as the three modulators α, λ, and A. Otherwise, all procedures followed appendix B in the study by Ahn et al. (2008; see Figure 2).

To explain further the removal of c, it is worth noting that the response-sensitivity parameter was introduced into behavioral models to resolve a problem of inconsistency. An explanation for this is as follows. Yechiam et al. (2005) suggested that:

“*the decision maker's choice on each trial is based not only on the expectancies produced by the decks, but also on the consistency with which the decision maker applies those expectancies when making choices.” (Yechiam et al., 2005, p. 975)*.

The final outcome defined the good and bad decks in the IGT; therefore, most behavioral models have been based largely on this basic assumption. Notably, while performing the IGT, participants typically do not realize the internal rules of the game during the initial stage. However, some participants will continue to select a single deck even as they are gaining insight regarding the good decks (Maia and McClelland, 2004) or will misinterpret the internal rule of the IGT, which may stand against the basic final outcome assumption (Lin et al., 2007; Chiu et al., 2008). Therefore, some research groups added the new parameter c in these models in order to solve this problem of inconsistency.

However, we considered that the incongruence between the basic final outcome assumption and modeling result can be solved by modulating the original parameters α, λ, and A. In fact, the value of parameters α and λ can directly modulate the effect of the monetary value in each gain-loss. Moreover, the value of parameter A can modulate the choice probability of consecutive trials through the influence of past experience. The modulations of these original parameters (α, λ, and A) can be used to observe the subjects' sensitivity to monetary value, the degree of skew for loss aversion, and the influence of past experience. In other words, if the simplified model does not increase the error rate (e.g., MSD) and decreases the calculation time, then this model may be considered much better than the original one (Busemeyer and Stout, 2002).

### Why adopt the MSD, not G^2^ scores?

Based on the statement by Ahn et al. (2008, p. 1387, Equation 9) for using the MSD and G^2^ scores as the criteria for evaluating these behavioral models, we decided to adopt the MSD but not the G^2^ scores as the evaluation criterion in this study. On the use of G^2^ scores, Ahn et al. (2008) state:

“*It is incorrect to simply use the product of the probabilities for choices across trials because independence does not hold.” (Ahn et al., 2008, p. 1399)*.

And on MSD scores, Ahn et al. (2008) state:

“*MSD scores are more intuitive for examining how good a model is in explaining overall choice patterns.” (Ahn et al., 2008, p. 1399)*.

Ahn et al. (2008) also pointed out the characteristics of the two evaluation indexes. Bearing all of this in mind therefore, we adopted the MSD scores as the index of parameter estimation to discover the optimal parameter sets.

## Results

In this study, we found a set of parameters and produced a simplified PU model to predict the choice behavior under uncertainty. The behavioral datasets were collected to serve as the benchmark data for comparisons with the modeling data. In addition, the key parameters α, λ, and A were systematically modulated and produced by the simulation data based on the PU models of Ahn et al. (2008). Specifically, the parameters were tested to screen out the best-fitted models as well as to determine the optimized range of parameters via MSD indexing. Based on the PU models, we found that there are some best-fitted models formed when some parameters are fixed. Notably, for the best-fitted models that we found, all three parameters were consistently nearly equal, with α ≈ 0; λ ≈ 1.3; and A ≈ 0.1. Obviously, the PU model in the present study was simpler than previous ones. However, the present PU model can produce optimized predictions for choice behavior under uncertainty, which is mostly consistent with the viewpoint of gain-loss frequency.

### Behavioral data

**Figure 3:**
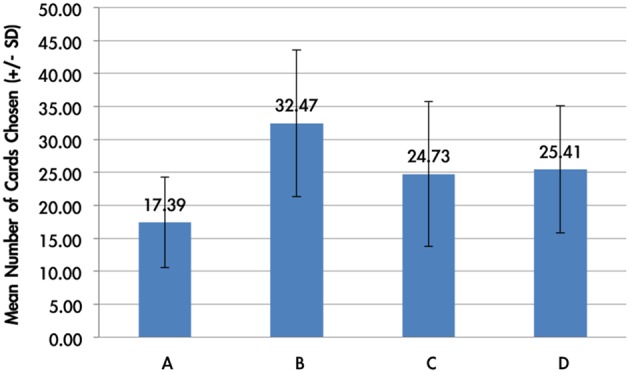
**Mean number of card selections in an average of 100 trials in the behavioral data**. The behavioral result showed that most subjects avoided the bad deck A, but preferred the bad deck B. The chosen number of bad deck B was nearly double that of bad deck A. However, participants preferred the good decks C and D only about the chance level (100/4 = 25).

The average card selection indicated that subjects preferred the good decks (C + D) nearly equally to bad decks (A + B; see Figure 3), which is inconsistent with the original finding from the IGT (Bechara et al., 1994). The two-factor repeated measurement ANOVA (final outcome vs. gain-loss frequency) was launched here to process the statistical testing. The testing result indicated no significant difference between the bad (A + B) and good (C + D) decks [*F*_(1, 144)_ = 0.23, *p* = 0.88], but the results showed a difference between the high-frequency (B + D) and low-frequency (A + C) gain decks [*F*_(1, 144)_ = 65.89, *p* < 0.001]. Furthermore, the interaction between the two factors (final outcome vs. gain-loss frequency) was also significant [*F*_(1, 144)_ = 66.28, *p* < 0.001]. However, detailed analysis of each of the two decks showed that the subject preferred to choose the bad deck B rather than the other three decks [*t*_A−B(144)_ = −12.59, *p* < 0.001; *t*_B−C(144)_ = 4.80, *p* < 0.001; *t*_B−D(144)_ = 4.93, *p* < 0.001] and that deck A was avoided compared to the other three decks [*t*_A−C(144)_ = −6.28, *p* < 0.001; *t*_A−D(144)_ = −7.50, *p* < 0.001]. Nevertheless, there are no significant differences between decks C and D [*t*_C−D(144)_ = −0.48, *p* = 0.63]. The present behavioral evidence confirmed the “prominent deck B phenomenon,” in which most normal decision makers were influenced by the frequent gain of the deck and the preference for the bad deck was difficult to inhibit by a few unexpected losses in the standard administration of the IGT (Wilder et al., 1998; MacPherson et al., 2002; Toplak et al., 2005; Fernie and Tunney, 2006; Chiu and Lin, 2007; Fernie, 2007; Lin et al., 2007; Martino et al., 2007;Takano et al., 2010; Upton et al., 2012; Steingroever et al., 2013; Worthy et al., 2013a).

**Figure 4:**
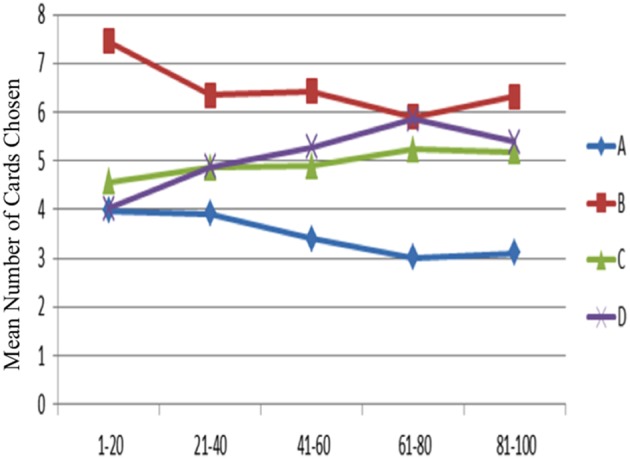
**Mean number of card selections in each block of 20 trials in the behavioral data**. Subjects preferred the bad deck B over the other three decks throughout most blocks. However, most subjects gradually avoided selecting the bad deck A from the beginning to the game end. Additionally, a slight ascending tendency for the good decks was observed from the first block to the end block, although statistical testing for the blocks in each deck was not significant.

The one-way ANOVA was applied to test the learning effect in each block of 20 trials (Figure 4). In detail, subjects' choice pattern for the bad decks A and B are descending over time, whereas the choice pattern for the good decks C and D are ascending. The learning-tendency analysis based on long-term outcome used the subtracted number between good decks and bad decks [(C + D)—(A + B)] in each block. The result indicated that the learning effect based on long-term outcome can be observed in this analysis [*F*_(4, 720)_ = 9.80, *p* < 0.001].

The learning-tendency analysis based on gain-loss frequency subtracted the number between frequent-gain (B + D) and frequent-loss (A + C) decks in each block. The result indicated that the learning effect based on gain-loss frequency cannot be observed in this analysis [*F*_(4, 720)_ = 0.60, *p* = 0.66].

However, detailed analysis of each deck in the blocks indicated that only three decks showed a significant learning tendency [*F*_A(4, 720)_ = 5.96, *p* < 0.001; *F*_B(4, 720)_ = 3.96, *p* < 0.01; *F*_C(4, 720)_ = 1.05, *p* = 0.38; *F*_D(4, 720)_ = 6.85, *p* < 0.001]. Furthermore, the *post hoc* analysis of each two-block in each deck demonstrated that the significant difference between each paired block existed mostly in deck A; in decks B and D there were only one and two significant differences between each paired block, respectively. The statistics are listed in detail in Table 2.

**Table 2:** **Summarized statistics after ***post hoc*** analysis of each two-block set for each deck**.

**Deck**	**A**	**B**	**C**	**D**
**Paired *t*-test for each two-block set**	**Sig**.	**Sig**.	**Sig**.	**Sig**.
B1-B2	1.000	0.072	1.000	0.218
B1-B3	0.264	0.129	1.000	0.007
B1-B4	0.002^**^	0.002^**^	0.745	0.000^***^
B1-B5	0.009^**^	0.059	0.976	0.003^**^
B2-B3	0.448	1.000	1.000	1.000
B2-B4	0.005^**^	1.000	1.000	0.086
B2-B5	0.020^*^	1.000	1.000	1.000
B3-B4	1.000	1.000	1.000	1.000
B3-B5	1.000	1.000	1.000	1.000
B4-B5	1.000	1.000	0.976	1.000

The values

* < 0.05;

** < 0.01;

*** *< 0.001 (Bonferroni Correction)*.

**Figure 5:**
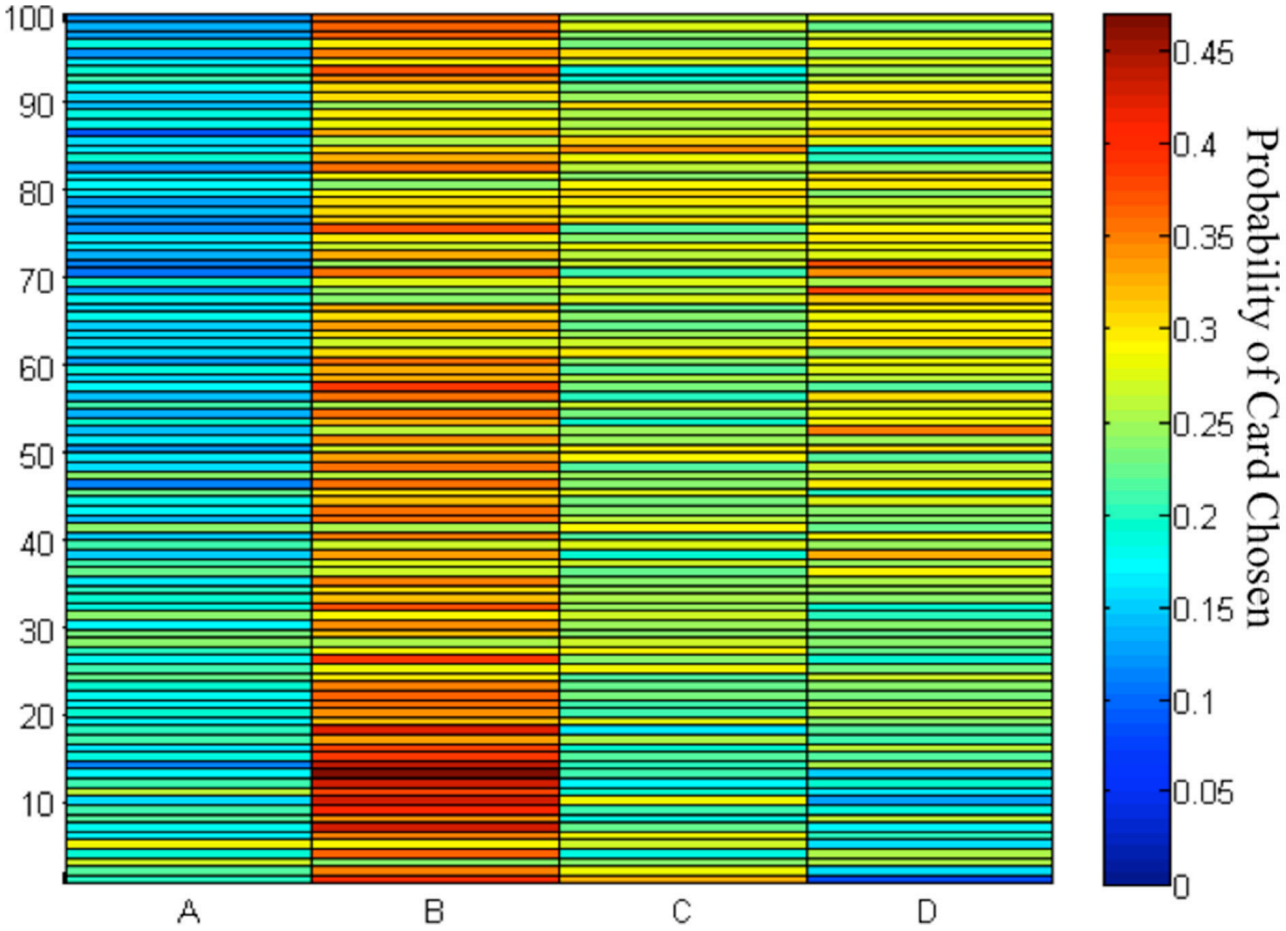
**Chosen probability maps for each deck across the 100 trials**. The red marks the high probability of card chosen and the blue marks the low probability of cards chosen. Of the 145 participants in the 100 trials of the IGT, most preferred to stay at decks B–D rather than deck A.

The choice probability of each deck in each trial showed that decks B, C, and D were preferred by the subjects throughout the game (Figure 5). The results confirmed the learning tendency for each deck (Figure 4).

### Simulation data

**Figure 6:**
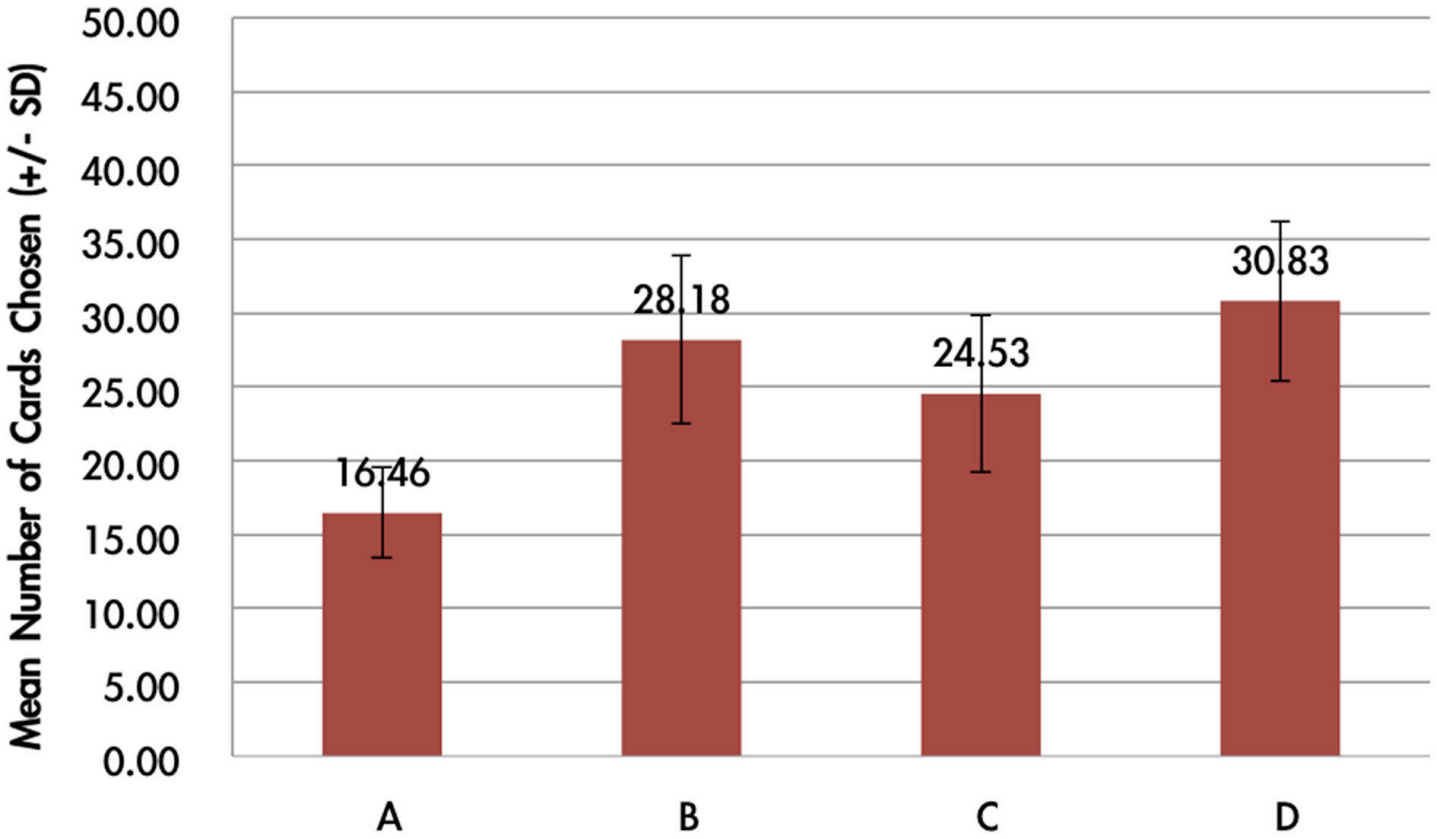
**Mean number of card selections in the simulation data**. Using optimized parameters simulated into the PU DEL model, the chosen number of decks B–D were larger than that of deck A. This simulation result is similar to the results of the participants in this study. The good deck D is widely selected, and the bad deck B was chosen slightly more frequently than the good deck C in the present simulation data.

**Figure 7:**
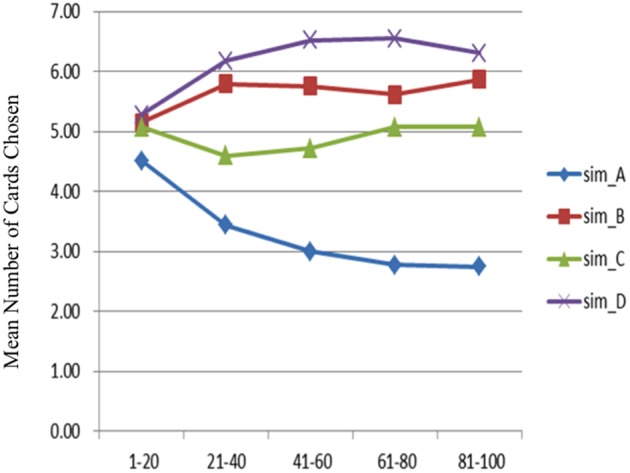
**Mean number of card selections in each block of 20 trials in the simulation data**. Putting parameters λ = 1.3, α = 0, and A = 0.1 into the PU model, the learning curve for deck A is descending, and decks B and D show a slightly ascending pattern from the beginning to the end of the game. The position of each curve in the simulation data is comparable to that done by the participants.

In the simulation data, the mean number of card selections showed that the number of cards chosen from the good decks (C + D) was nearly equal to the number chosen from the bad decks (A + B; Figures 6, 7). The two-factor repeated measurement ANOVA (final outcome vs. gain-loss frequency) was used to further demonstrate the statistical result under the simulation level. The results showed significant differences between the bad (A + B) and good (C + D) decks [*F*_(1, 144)_ = 135.85, *p* < 0.001]. On the other hand, a significant effect was also observed between the high-frequency (B + D) and low-frequency (A + C) gain decks [*F*_(1, 144)_ = 312.47, *p* < 0.001]. Additionally, the interaction of the final outcome and gain-loss frequency was significant [*F*_(1, 144)_ = 34.32, *p* < 0.001]. However, a paired-t analysis showed that differences between each two decks were all significant [*t*_A−B(144)_ = −19.37, *p* < 0.001; *t*_A−C(144)_ = −15.86, *p* < 0.001; *t*_A−D(144)_ = −25.38, *p* < 0.001; *t*_B−C(144)_=4.63, *p* < 0.001; *t*_B−D(144)_ = −3.44, *p* < 0.001; *t*_C−D(144)_ = −8.26, *p* < 0.001]. The present data confirmed that the “prominent deck B phenomenon” is reproduced under the simulation environment. According to learning curve analysis (Figure 7), the choice patterns for the bad deck B and good deck D seem to rise over time, whereas the choice pattern of the good deck C stays consistent while that of bad deck A decreases.

### Comparison between behavioral and simulation data

A comparison of the behavioral and simulation data (the group data with the smallest MSD) shows that no significant difference was observed [*F*_(1, 288)_ = 1.06, *p* = 0.30]. In short, the simulation data was similar to the actual chosen pattern of these participants.

### Simulation result evaluation

**Figure 8:**
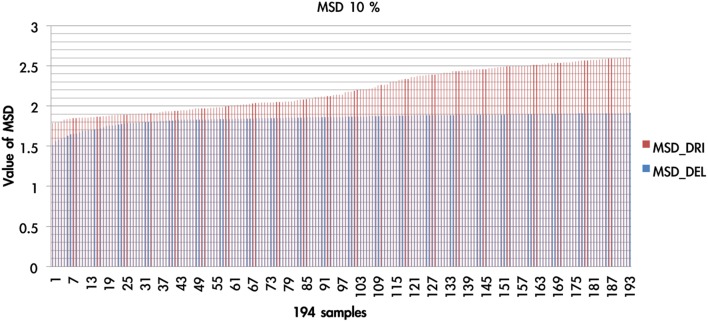
**The MSD of DRI and DEL in first 10%**. Based on the simulation results of the DRI and DEL models, we took the first 10% (194 simulation cases) of the simulation samples that possessed the smallest MSD value. The red line represents the MSD value (by simulation) of the DRI model, and the blue line marks that of the DEL model. The MSD of the DEL model is smaller than that of the DRI model. The DEL model possessed a better goodness-of-fit than the DRI model.

There were 1936 optimal MSD parameter sets after the parametric estimation (α: 11 values (per 0.1): range [0–1]; λ: 16 values (per 0.1): range [1–2.5]; A: 11 values (per 0.1): range [0–1]). First, we presented the data using an ascending sequence to show the situations of 1, 5, and 10% MSD distribution. Figure 8 shows the first 10% MSD error distribution. The result demonstrates the number on the horizontal axis to be positively correlated with the MSD error on the vertical axis. Therefore, this observation confirms the high reliability of these parameter sets (α, λ, A).

**Figure 9:**
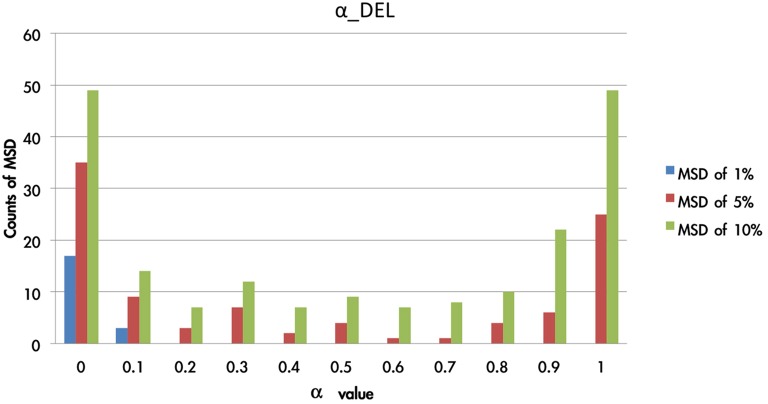
**Counts of smallest MSD value when modulating the α value based on the DEL model**. Based on the DEL model, this test modulated the value of α from 0 to 1 and processing with 1936 simulations. The MSD values of the 1936 samples were listed in ascending order. Accordingly, in the first smallest MSD conditions (ranked as 1, 5, 10%), the counts of variant α value were calculated. Notably, when the α value was nearly equal to zero, there was the largest number of smallest MSD in the collection. In other words, the conditions with the smallest MSD were observed mostly when the α value was close to zero. The parameter α can be changed as a constant in the best-predictive DEL models.

**Figure 10:**
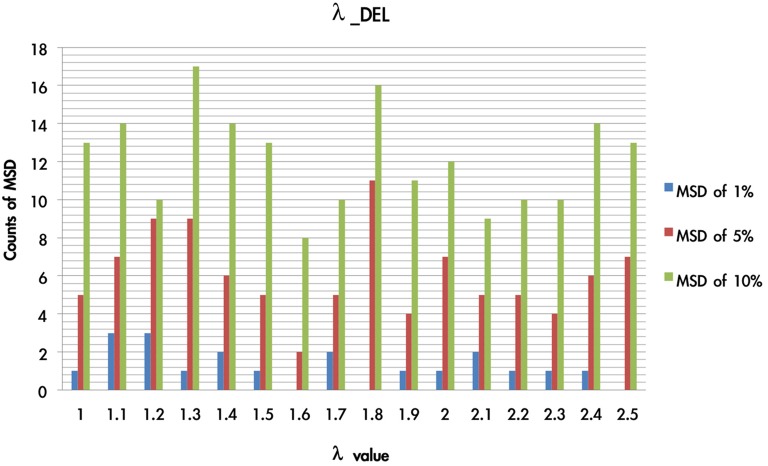
**Counts of smallest MSD value when modulating the λ value based on DEL model**. Based on the DEL model, this test modulated the value of λ from 1 to 2.5 and processing with 1936 simulations. The simulation trials were ranked according to the MSD value in ascending power. In the three smallest MSD conditions (1, 5, 10%), when the λ value was located on 1–1.5 there are stable numbers of the smallest MSD in the distribution. This indicates that in the DEL model, λ can be made a constant by giving it the value of 1.3.

**Figure 11:**
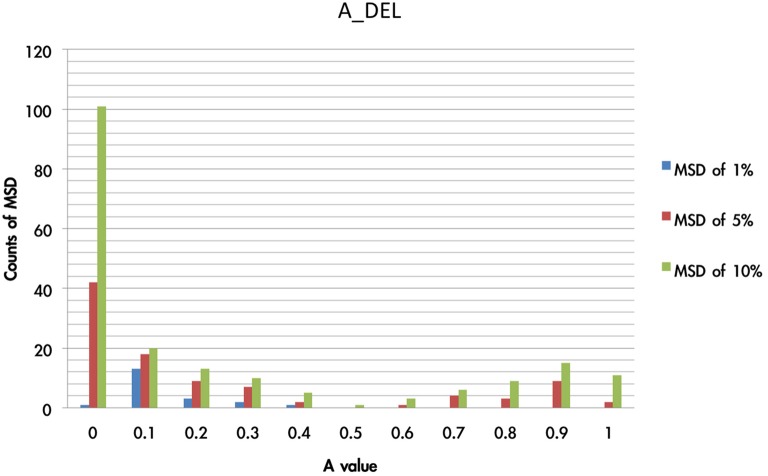
**Counts of smallest MSD value when modulating the A value based on DEL model**. The present test modulated the value of A from 0 to 1 and processing with 1936 simulations based on the DEL model. The simulation results were listed with regard to the MSD value. Here we demonstrated that in the three collections (1, 5, 10%) of smallest MSD values, the A value close to zero has the largest number of smallest MSD. This indicates that in the best-fit model (e.g., DEL), the parameter A may be fixed to a constant (close to zero) rather than a variable, which represents the ineffective influence of past experience.

We overlaid the MSDs of the DRI and DEL models in Figure 8. The result showed that the DEL model was more accurate in making prediction than the DRI model. This finding is consistent with the previous observation of Ahn et al. (2008, p. 1392; Table 6) which showed that the MSD of the DEL model was relatively small in comparison with the MSD of the DRI model. The following figures demonstrate the number of value distributions (0 < α < 1; 1 < λ < 2.5; 0 < A < 1) under three MSD conditions (1, 5, 10%) for each parameter (α, λ, A) in the DEL model (Figures 9–**11**).

Figure 9 shows that the 1% MSD is clearly allocated mostly in the low α-value section (e.g., 0 and 0.1). This impact of gain-loss value is relatively restricted or vanishing for decision makers. When α is close to zero, x(t) is almost close to 1. This indicates the influence of the gain-loss frequency and the impact of λ and A. Based on the three hierarchies of MSD (the error rates from 1 to 10%), the small value of α possessed a relatively high reliability.

As shown in Figure 10, the simulation test demonstrates that when the λ value was in the present range (1 < λ < 2.5), the MSD distribution patterns (MSD of 1–10%) did not change significantly. When the value of α was close to zero (MSD of 1%), the λ value influenced the fluctuation of MSD value to a lesser degree. Furthermore, when the α value was equal to 0, the function of x(t) was equal to 1, and the weight effect of λ disappeared. In fact, the probability of loss trial in the IGT was only 20%. As the probability of choosing loss trial is relatively small, the appearance frequency of the λ value has an averaged distribution globally.

Additionally, the value of A influenced the consecutive trials; namely, the acquisition of strategy learning in an abstract manner. For instance, if the A value is small, the effect of influencing the consecutive trial by the previous gain-loss experiences is relatively small. In Figure 11, it can be observed that the A value is located in a relatively small range of the MSD.

## Discussion

The empirical results of this study replicated the “prominent deck B phenomenon” in the IGT and demonstrated that most subjects preferred the bad deck B and good decks C and D rather than the bad deck A in the standard administration of IGT (see Figures 3–5). However, various research groups have made this observation on the behavioral level over the past decades (Wilder et al., 1998; Takano et al., 2010; Upton et al., 2012; Steingroever et al., 2013; Worthy et al., 2013a,b). The present modeling study indicated that some parameters in the PU model may be ineffectual in predicting the choice behavior in IGT. Therefore, we suggest that the Ahn et al. (2008) PU model is not the optimal one and that there should be some room for modification.

### The simulation based on the mean number of card selections

**Figure 12:**
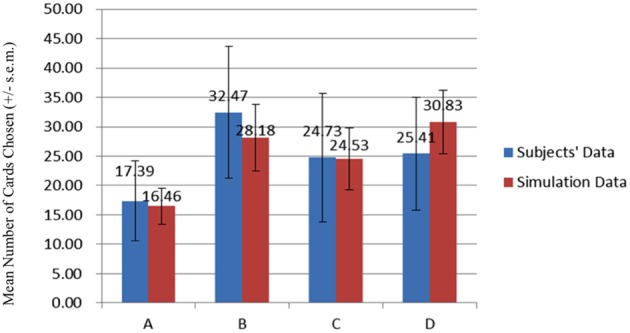
**Comparison of two datasets for average card selection (for the first 100 trials)**. Comparing the data of the subjects and the simulation data, we observe that the number of chosen cards from decks B and D was higher than that of the other two decks. The high selection of these two decks suggests high frequent gain to be the critical factor in choice behavior under uncertainty. Furthermore, the bad deck A was consistently the least chosen in the behavioral and simulation data. This observation is congruent with those of most previous IGT studies.

According to the simulation result of the choice pattern in each deck, deck A is relatively lower than the other three decks (Figure 12). Decks B, C, and D have a similar mean number of card selections. This choice pattern (A < B, C, D) existed not only in the empirical data, but also in the simulation data. The simulation result is similar to the empirical observation of the IGT choice behavior. We found that in the gain-loss structure of the IGT, two main factors, monetary value and gain-loss frequency, correlated highly with the present choice pattern. For instance, in a circle of 10 trials, decks B and D have relatively high frequency gains; for example, nine gains and one loss (Wilder et al., 1998; Worthy et al., 2013a,b; Seeley et al., 2014). If the monetary value is controlled between the two decks, the two decks will have the same gain-loss structure. The choice pattern of simulation data shows that decks B and D have a similar number of choices when the α value is close to zero. Monetary value has less influence in this condition (Lee et al., 2014).

### Observation of the learning processing

The empirical and simulation data consistently demonstrated the learning curve of deck A to be gradually descending (Figures 4, 7). On the other hand, both behavioral and simulation findings showed similar ascending choice patterns for decks C and D. However, some differences between the behavioral and simulation data for deck B was observed, which may have arisen from some limitations in the present models. In fact, Ahn et al. (2008) mentioned that the best model (DEL) in their IGT and SGT simulation study could make enhanced predictions for global choice patterns (long-term predictions) but not for learning processing (Ahn et al., 2008, 2014).

Additionally, based on the viewpoint of gain-loss frequency, the ascending curves of decks B and D may be due to the decreasing influence of monetary value. Moreover, the location of the learning curve of deck C in the middle of the four curves may be due to the deck's occasional draws (for example, “+50, −50” in some trials) and small gains from the viewpoint of net-value calculation (Chiu and Lin, 2007; Chiu et al., 2012).

**Figure 13:**
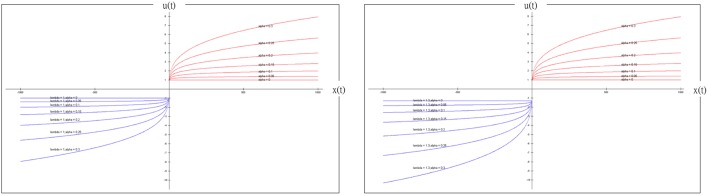
**Prospect utility function and simulated gain-loss frequency effect (λ = 1 (left panel); λ = 1.3 (right panel))**. According to the definition provided by Ahn et al. (2008), α is defined as the shape of the utility function and λ as the response to the effect of loss aversion. When α or λ is adjusted in the PU function, we observed little loss-aversion effect. Notably, the present study indicated that both α and λ might not be the variables that shape the selection pattern of decks A, B, C and D. Particularly, when λ = 1, the loss-aversion effect is no longer present in this figure. According to our simulation result, the optimized α value is nearly equal to zero, the PU function only represents the effect of gain-loss frequency and the effect of value is greatly diminished. More specifically, when the optimized λ value is nearly equal to 1 and α close to zero in this simulation, the value function is similar to the Heaviside (step) function. This observation implies that the effect of insensitivity to value is actually the same as the effect of sensitivity to gain-loss frequency (Lin et al., 2007; Chiu et al., 2008). The present simplified PU model mostly represents the adoption of a gain-stay loss-shift strategy under uncertainty. This finding may explain the “prominent deck B phenomenon” for healthy groups in the growing number of recent IGT studies.

The model in the present study combined the PU function and delta learning model and undoubtedly created a hierarchical influence. The order of influence could be α to A (positive net value) or α, λ to A (negative net value). Based on this observation, α is a powerful parameter for modulating the model and predicting the participant's behavior. Conversely, when α is fixed, λ and A have less influence in mediating the model. Therefore, the value of α obviously determines the effect significantly. On the other hand, the simulation result of α (Figure 9) demonstrated the model to be insensitive to value change, but it correlated increasingly to the gain-loss frequency effect (Figure 13). Nevertheless, based on the behavioral result (Figure 4), the selection of bad deck B gradually and unsteadily decreased. This may imply that the largest loss of deck B truly does influence choice behavior; thus, the small α value (0) of the simulation and the λ value (1.3) may not totally reflect all situations.

Over the past decade, studies of IGT modeling have evolved from the linear EU model (Busemeyer and Stout, 2002) to the non-linear PU model (Ahn et al., 2008). These models aim to quantify behavioral impact by monetary value. Notably, the PU model possessed unequal valence and value function between gain and loss; namely, unbalanced marginal effect in gain and loss conditions. However, many components from the input (perception) to the output (decision making) may influence the behavioral results. For example: visual fields, figure and character distinction, the ability to integrate information, memory encoding, and retrieval, comprehension, logical reasoning, and decision drivers may be latent causes that also influence choice behavior. The present PU model considered only a partial set of relevant variables when predicting the decision behavior under uncertainty. There may be better and more simplified models using dynamic-change parameters.

## Conclusion

Based on PT theory and the study by Ahn et al. (2008), we found a simpler model of IGT behavior in the present study. Over the years, many IGT modeling studies have suggested that the PU model (Ahn et al., 2008) is better than the EU model (Busemeyer and Stout, 2002) for predicting choice behavior under uncertainty because the PU model considers the distinct influences of gain and loss. However, we considered that some parameters in the PU model may be ineffective and render this model suboptimal. In this study, we provided a method of model testing by modulating some key parameters (α, λ, and A) in the PU model. The findings from the model testing demonstrated that these parameters (α, λ, and A) possessed hierarchical influences and specific optimized ranges in the PU model. By setting α ≈ 0; λ ≈ 1.3; and A ≈ 0.1 as the optimized parameters of the simulation, the modified PU function (u(t)) can be calculated as follows:

$$u(t)=\{\begin{array}{c} x{\left(t\right)}^{\alpha }\\ -\lambda |x(t){|}^{\alpha } \end{array}=\{\begin{array}{c} 1,\;if\;x(t)\geq 0\\ -1.3,\;if\;x(t)<0 \end{array}$$

As α is approaching zero, the shape of this function is similar to a Heaviside (step) function (see Figure 13).

Combined with this result, we suggest a simplified model as follows:

$$\begin{array}{l} {\text{E}}_{\text{j}}(\text{t})=\;{\text{E}}_{\text{j}}(\text{t}-1)+\text{0}.\text{1}\cdot \delta_{\text{j}}(\text{t})\cdot [1-{\text{E}}_{\text{j}}(\text{t}-1)],\text{ if x}(\text{t})\geq 0;\\ {\text{E}}_{\text{j}}(\text{t})=\;{\text{E}}_{\text{j}}(\text{t}-1)+\text{0}.\text{1}\cdot \delta_{\text{j}}(\text{t})\cdot [-\text{1}.\text{3}-{\text{E}}_{\text{j}}(\text{t}-1)],\text{ if x}(\text{t})<0. \end{array}$$

Further, we conclude that the change in some parameters (e.g., λ and A) may be powerless in influencing the models when α approaches zero. This model testing shows that the PU model may need further simplification for it to be optimized. The simulation of this simplified model implied that decision makers were sensitive to gain-loss frequency rather than the long-term outcome. The modified model may possess better predictors for clinical categorization and distinguishing between normal subjects and neuropsychiatric patients. However, the present study determined a set of the three fixed values for α, λ, and A only by analyzing a specific dataset of IGT experimental data. To make this dataset of estimated values applicable to a wider range of IGT and SGT experiments, more data from different experimental sets would be needed. Supposing the fitting values for α, λ, and A could be converged to an acceptable range across a sufficient number of experiments, this simplified model may turn out to be a better explanation of choice behavior under uncertainty.

## Author contributions

CL, YL, and YC contributed to the conceptual innovation and literature review and the three authors contributed equally to this study. JH provided some valuable concepts. YL and TS were responsible for subject recruitment and behavioral data collection. YL contributed to the programming of models and simulation data processing. YL, TS, and CL provided the statistical analysis. YC, YL, and CL worked on the data interpretation and developed the manuscript. Also, JH provided some discussion on refining the manuscript. YC, YL, and CL set up all experimental conditions and arranged all behavioral and simulation circumstances for this study. YC, JH, and CL finalized all revisions with YL and TS.

### Conflict of interest statement

The authors declare that the research was conducted in the absence of any commercial or financial relationships that could be construed as a potential conflict of interest. The reviewer PM and handling Editor declared their shared affiliation, and the handling Editor states that the process nevertheless met the standards of a fair and objective review.

## Abbreviations

DEL: delta learning rule
DRI: decay reinforcement learning rule
EU: expected utility
IGT: Iowa Gambling Task
MSD: mean square deviation
PU: prospect utility
PT: prospect theory.
